# The observation of starch digestion in blue mussel *Mytilus galloprovincialis* exposed to microplastic particles under varied food conditions

**DOI:** 10.1371/journal.pone.0253802

**Published:** 2021-07-06

**Authors:** C. J. O’Brien, Helen C. Hong, Emily E. Bryant, Kwasi M. Connor

**Affiliations:** 1 Department of Biology, California Lutheran University, Thousand Oaks, California, United States of America; 2 Department of Biology, University of California, Irvine, California, United States of America; VIT University, INDIA

## Abstract

Microplastic continues to be an environmental concern, especially for filter feeding bivalves known to ingest these particles. It is important to understand the effects of microplastic particles on the physiological performance of these bivalves and many studies have investigated their impact on various physiological processes. This study investigated the effects of microplastic (10 μm) on digestive enzyme (amylase) activity of *Mytilus galloprovincialis* at 55,000 and 110,000 microplastic particles/L under laboratory conditions. Additionally, our study measured the expression of an isoform of Hsp70 in the gills to assess whether or not these particles may cause protein denaturation. Results revealed that this regime negatively affect the ability of *M*. *galloprovincialis* to digest starch under high food conditions but not low food conditions. Exposure to extreme levels of microplastic raised amylase activity. Furthermore, Hsp70 transcript abundance was not elevated in treatment mussels. These results show that mussels may be resilient to current microplastic pollution levels in nature.

## Introduction

Microplastics are manmade polymers that are ubiquitous throughout the world. Investigators have identified microplastics in artic snow [1], the deep sea [2], fruits and vegetables [3], human stool [4], and other macro and microenvironments [5]. As new investigations of plastic debris emerge, it is clear that microplastic abundance in the environment is a phenomenon that poses negative health consequences for all organisms on the planet. Detrimentally, plastic production has no indication of slowing down and is expected to double within 20 years [6].

Plastic breaks down by UV radiation, chemical degradation, wave mechanics, and grazing marine life [7], but does not fully degrade. Plastic spheres or fragments with a diameter/length < 5mm are considered microplastics [8, 9]. Several studies have shown that microplastics pollute the ocean through a variety of anthropogenic processes [10] and the scientific community is in agreement that these particles will have long-term consequences for marine life.

Microplastics have been found within tissues and organs of a variety of wild and cultured marine invertebrates including zooplankton, polychaetes, crustaceans, and bivalves (reviewed in Phuong et al.; 2016) [8]. Filter-feeders, such as sessile bivalves, are the most susceptible to small, floating pieces of plastic [11]. During active feeding, bivalves can continuously pump and filter seawater through coordinated action of cilia localized at the gill epithelium surface, trapping anything of an appropriate size that comes in contact with gill [12]. The concentration of microplastic exposure and feeding behavior of bivalves is highly dependent on environmental conditions; this varies widely throughout the ocean. Environmental reports of ocean microplastic pollution revealed volumetric concentrations that ranged from 0.005 to 9,200 particles/m^3^ (5x10^-6^ to 9.2 particles/L) [8]. Heavily polluted coastal regions can exceed 100,000 particles/m^3^ (100 particles/L) which has the potential to negatively impact the coastal environment [13]. To this end, Severini et al. (2019) [14] observed microplastics in the gut of oyster *Crassostria gigas* residing in an estuary with a maximum microplastic concentration of ≈ 800 particles/L.

Microplastic concentrations within the ocean are predicted to increase 50-fold by 2100 [15], while storm events can increase abundance up to 40-fold in nearshore systems [16]. Hence, benthic organisms may be subjected to concentrations in the tens of thousands particles per liter in the future depending on region, microenvironment, and season. In this context, a series of laboratory studies have been conducted in attempts to unveil the negative effects of microplastics (< 5 mm diameter/length) on physiological performance in filter-feeding bivalves. Results of these studies show elevated hemocyte mortality, reactive oxygen species production [17], increased antioxidation activity [18], lowered respiration rates [19], decreased oocyte number [17], cellular damage, alterations in gene expression [20], inflammation response, lysosomal membrane destabilization [21], reduced filtering activity [22, 23], and more. Most studies on physiological performance of organisms exposed to microplastics subjected individuals to concentrations (tens of thousands particles/L to billions of particles/L or high w/v ratios) well above published natural offshore surface water conditions [24]. However, they are important because they can help to predict how these organisms may cope with future concentration levels.

The digestive gland of *Mytilus* species is the primary organ for digesting organic polymers and acquiring energy; therefore, necessitating the need to understand how it is impacted when mussels are subjected to microplastic contamination. Previous reports revealed that microplastics invade the digestive gland in laboratory-exposed organisms at high concentrations (above the reported sea surface measurements) [21, 25]. Hence, a study by Van Cauwenberghe et al. (2015) [26] revealed no effect of high concentrations of polystyrene beads on cellular energy allocation in the digestive gland of *M*. *edulis*, suggestive of limited environmental related stress on the organ. Although no effect was observed, Van Cauwnberghe et al. (2015) is not informative of the effects of microplastic exposure on digestive gland function. In the current study, we investigated whether microplastics affect the function of the digestive gland in *M*. *galloprovincialis* by measuring amylase enzyme activity in mussels exposed to microplastic particles under two food concentration levels. Amylase is secreted into the gut by the digestive gland and hydrolyses α-1,4 of glycosidic linkages of starch. It is a robust marker of digestive gland function because it is the highest-expressed digestive enzyme in *Mytilus* [27]. Because exposure of mussels to high concentrations of microplastics leads to the presence of microparticles within the digestive gland [26], we predicted that digestive enzyme activity would decrease following exposure to microplastic at these extreme concentrations. However, the negative effects would be less severe in well-fed mussels. Additionally, our study measured the expression of an isoform of heat shock protein in the gills to assess whether microplastic causes a protein denaturing stress-response in exposed organisms.

## Materials and methods

### Acclimation

Mussels of 5–7 cm length and 21 months of age were donated from Catalina Sea Ranch Aqua Farm (Long Beach, CA). Mussels were evenly distributed into two 25-gallon tanks and acclimated for 4 weeks in artificial 35 ppt seawater using Instant Ocean^™^ (Instant Ocean Spectrum Brands, Blacksburg, VA) at 17°C. To simulate the two levels of energy balance that occurs from tidal variation or seasonal changes in food abundance, two food regimes were implemented using Shellfish Diet 1800^™^ (Reed Mariculture, Campbell CA) based upon previous reported protocols in Connor et al. (2016) [27]. The low and high concentrations of food in the experimental tanks were ~8 and ~15 mg/L of particulate organic matter respectively. Shellfish Diet 1800^™^ consists of Isochrysis sp. 40.0%, Pavlova sp 15%, Thalossiosira weissflogii 20.0%, and Tetraselmis sp. 25.0% dry weight. After the acclimation period, 24 mussels from each food regime were split evenly into one control (no plastic) and two treatment groups (low and high plastic), and placed into 1,000 mL beakers (four mussels per beaker) filled with seawater. Air was introduced from the bottom of each vessel to maintain food and plastic suspension. Food was introduced to vessels daily at corresponding low and high acclimation concentrations. In addition, polystyrene spheres (10 μm) (SIGMA-72986) were also added to vessels daily to create low and high microplastic concentrations of 55,000 and 110,000 particles/L respectively. The water was changed each day to maintain consistent concentrations of food and microplastics spanning a seven-day period.

### Enzyme activity

Protocols were similar to those used in Connor et al. (2016) [27]. The Somoygi-Nelson method was used to measure amylase activity in each mussel. Digestive glands were weighed and homogenized in malic acid buffer (pH 6.55). Substrate was combined with buffer and homogenate, then incubated at 17°C for 30 minutes. After incubation, Somoygi-Nelson reagent A was added, followed by reagent B. The absorbance was measured by a spectrophotometer at a wavelength of 655 nm. Amylase activity was determined using a standard curve. Mass-specific enzyme activities are expressed in U (1 μmol reducing sugar liberated per minute) per gram wet weight digestive gland tissue.

### Gene expression

We assessed whether microplastics caused protein denaturing in gill tissue. Chaperone gene *Hsp70B2* has been shown to respond to toxins and heat in previous studies of *Mytilus californianus* [28–31]. We first performed a proof-of-concept experiment to show that *Hsp70B2* responds to heat stress in *M*. *galloprovincialis*. Subsequently, we assessed variation in *Hsp70B2* between low-food-control (LFC) and low-food/low-plastic (LFLP) groups. We chose the low plastic groups because the concentration is still beyond current real-world conditions. We used BLAST to align an EST sequence in *M*. *californianus* (GenBank: ES735872.1) previously shown to respond to temperature [27], with the *M*. *galloprovincialis* transcriptome (90% identity to transcript GenBank: GAEM01001309.1). Resulting primers were FWD: TACCTGGTCGTTGGCTATG and REV: CGACGATTGAGAGGGCAAG. Tubulin a was used as a reference gene (FWD: CTTCGGTGGTGGTACTGGAT and REV: AGTGCTCAAGGGTGGTATGG). For the proof-of-concept experiment, we assessed heat-shock protein expression levels in mussels subjected to conditions observed in the field in Southern California. This includes thermal warming with valves closed during low tide, cool conditions with valves closed, and cool conditions with valves open. We placed mussels in two plastic sealed bags (N = 3) to separate individuals from oxygen and induce valve closure. The bags of mussels were placed in water baths set to 17°C and the *Hsp70B* expression levels were measured for 4 samples from control (low-food) and treatment (low-food/low-plastic groups). RNA was extracted from samples using TRIzol (QIAGEN), followed by reverse transcription (Primescript^™^, Takara). The resulting cDNA (1 μl) was used in a RT-qPCR reaction (iTaq, BioRad), and amplified with a Roche thermal cycler and the designed primers. Relative expression was measured and calculated using the Δ-ΔCt method.

## Statistics

The method described in the study by Carling (2000) [32] was used to detect outliers with R-package Rallfun [33]. A total of five outliers were removed with this method. Subsequently, a two-way ANOVA was used to test the null hypothesis: that there is no difference in amylase enzyme activity across food-level, microplastic concentration, and their interaction. The means of each treatment were compared with Tukey’s test. Finally, a *t*-test was used to compare the means of *Hsp70B* transcript abundance between LFC and LFLP groups.

## Results

**Fig 1:**
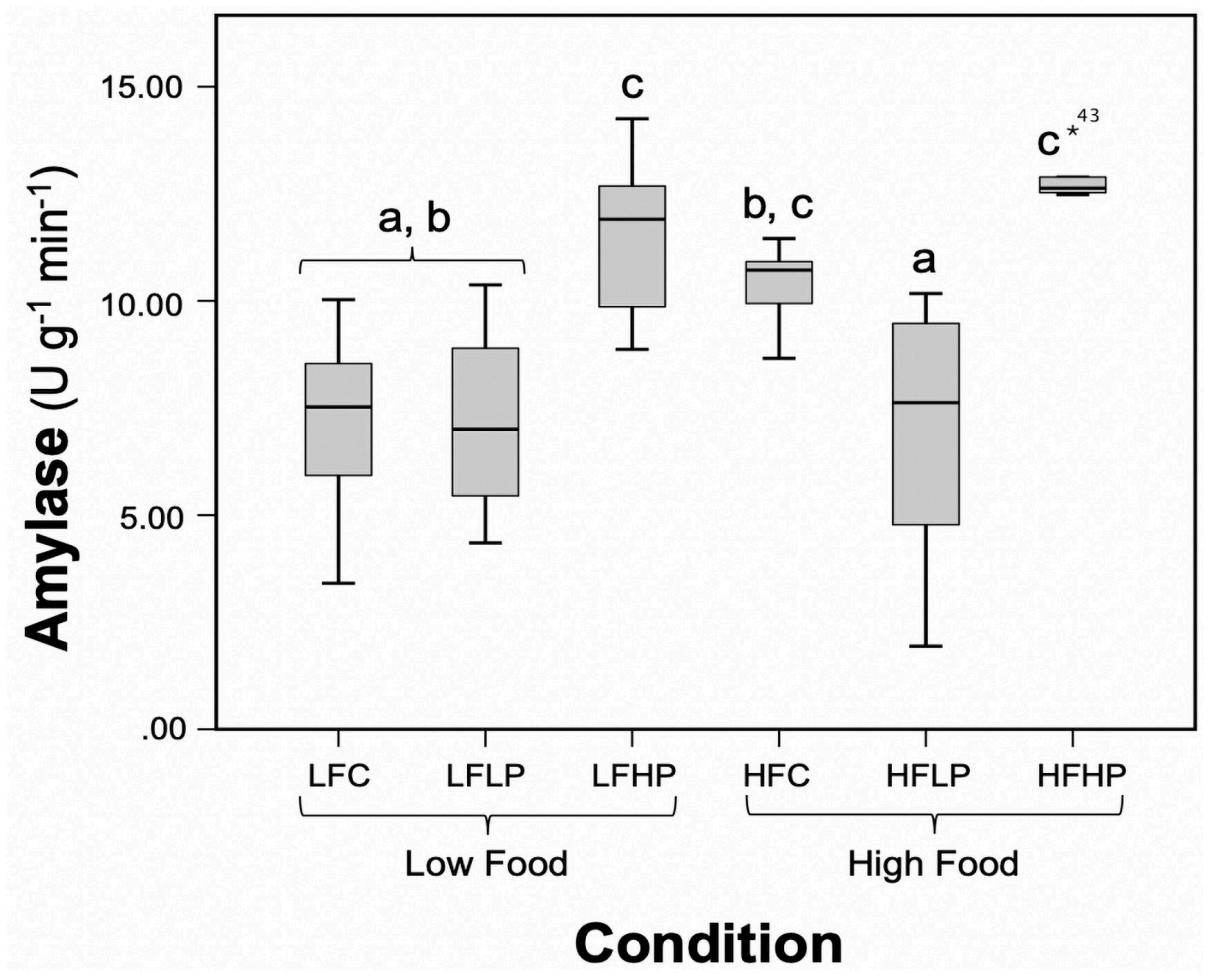
Variation in amylase activity in mussels between treatment groups. Tukey’s test was used to statistically illuminate variation, noted by letters. LFC = Low Food Control, LFLP = Low Food Low Plastic, LFHP = Low Food High Food Control, HFLP = High Food Low Plastic, HFHP = High Food High Plastic.

**Fig 2:**
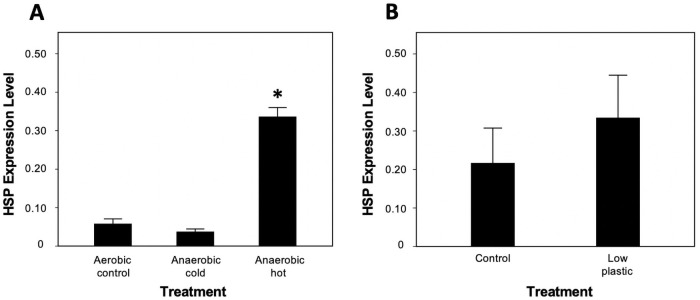
Heat shock protein-70B expression levels in A) heat experiment N = 3 and B) plastic experiment N = 4. In the heat experiment (proof of concept of stress response), mussels were exposed to aerobic/anaerobic conditions at cold and hot temperatures. In the microplastic experiment mussels were subjected to no (control) and low plastic particles under low-food conditions.

A two-way ANOVA revealed significant differences between groups (*P = *0.001; Table 1; S1 File). The main effect of plastic was shown to vary between groups (*P <* 0.001; Table 1). The highest enzyme activities occurred under low-food/high-plastic (LFHP) and high-food/high-plastic (HFHP) groups, which were 11.44 and 12.86 U g^-1^ min^-1^ respectively. This suggests an enhancing effect of exceedingly high microplastic presentation on enzyme activity. The three lowest values occurred under the high-food/low-plastic (HFLP), LFC, and LFLP groups, which were 6.98, 7.18, and 7.18 U g^-1^ min^-1^ respectively. The high-plastic treatment groups were greater than all other groups except for high-food control, which had only a slightly lower mean value (Fig 1; S1 File). The mean activity level of the low-plastic group was significantly lower (33%) than the control under high-food acclimation, suggesting a negative effect of microplastic under certain laboratory conditions (Fig 1). The expression of *Hsp70B* was approximately 7-fold higher during warming than when exposed to cool conditions in the proof-of-concept experiment (Fig 2). However, *Hsp70B* transcript abundance was not elevated in the low-food-low-plastic abundance treatment (Fig 2).

**Table 1:** Analysis of variance between experimental groups of mussels subjected to different food levels and microplastic. *P* values <0.05 were considered significant.

Tests of Between-Subjects Effects				
Source	df	MS	*F*	*P*
Intercept	1	3675.077	574.125	0.000
Food level	1	13.089	2.045	0.160
Plastic	2	74.859	11.695	0.000
Food level ⨉ plastic	2	4.979	.778	0.466
Error	41	6.401		

## Discussion

The effect of microplastics on the digestive system of *M*. *galloprovincialis* under laboratory conditions was assessed under varied plastic concentrations and food conditions in order to explore future implications for mussel health when they are exposed to elevated plastic pollutants. We subjected mussels to particle concentrations shown to invade gut cavities and expose the cells that line them [34]. Amylase was negatively affected by exposure to 10 μm microplastics but not consistently across food regimes. Enzyme activity was negatively affected under high-food conditions only. Therefore, it is possible that microplastic contamination could affect nutrient acquisition in *M*. *galloprovincialis* under certain environmental conditions in nature. Yet, the mechanisms of this reduction in activity are unknown. To this end, Wang et al. (2020) [35] showed a decrease in digestive gland amylase activity in a small sample size of hard-shelled mussel *M*. *coruscus*, exposed to > 10,000 particles/L of 2 μm-sized polystyrene spheres. Hard shelled mussels (*M*. *coruscus* and *M*. *californianus*) and blue mussels (*Mytilus edulis* complex), including *M*. *galloprovincialis* are spatially segregated in nature. Hard-shelled mussels flourish in high flow wave-exposed regions of shore and blue mussels are constrained within slow-flowing bays [36]. It is possible that these environments could have selected for different feeding and digestive mechanisms amongst species within *Mytilus*. More studies of both lineages are necessary to make comparisons of their responses to microplastic exposure. Furthermore, the sphere size of microplastic used in that study could have caused a toxicological related response. For example, Paul-Pont et al. (2016) [17] showed digestive gland tissue degeneration following exposure (2,000,000 particles/L) to particles 2–6 μm in size. Also, particle size determines the rate at which microplastics translocate from the gut to the circulatory system. Browne et al. (2008) [34] found that particles 3 μm in size accumulated in the hemolymph more than those that were 9 μm. The authors suggest that particles within the circulatory system present a hazard to all tissues within the organism. This finding is in agreement with microplastic particle size selectivity revealed in Mytilus [37]. In conclusion, particle size is just one of many variables that make comparisons between physiology-based studies difficult.

Amylase activity was negatively affected by plastic particles in mussels exposed to 55,000 particles/L in the high food group. Other studies have also revealed that the ingestion of microplastics > 2 μm perturb digestive gland tissue under laboratory conditions. For example, Von Moos et al. (2012) [21] observed in *M*. *edulis*, plastic particles (1,000,000 particles/L) contained within the lysosomal system of digestive gland cells and a simultaneous steady increase of white blood cells (eosinophil granulocytes) with rising particle exposure. Furthermore, microplastics of any size can adsorb organic pollutants from the environment or be coated with a variety of possible toxins during manufacturing [38]. Alternative to the inference that toxicity led to reduced activity in the high-food environment, subjecting mussels to microplastics could have reduced filtration rate, which in turn could negatively affect the amount of food delivered to the mouth and ingestion rates. Filtration rate is determined by valve closure and gill function. We did not observe toxicological effects at the molecular level using *Hsp70B* as a marker, suggestive of normal gill function. However, more gene expression and histology studies are necessary to assess whether gill perturbations by 10 μm microplastics could lead to lowered ingestion rates of food. Lastly, well fed mussels may have displayed reduced enzyme activity due to microplastic exposure as a result of nutrient balancing processes [39]. The demand for nutrients may have been depressed in well-fed mussels, which led to lowered digestive investment: a physiological process which has been shown in other organisms [39, 40]. More work on nutrient balancing process modulated by digestive enzyme activity in *Mytilus* is greatly needed in order to resolve these questions.

In the present study, microplastic and algal cells of the food were similarly sized and added simultaneously to treatment vessels and this could have affected enzyme activity. Amylase activity was found to be higher in mussels exposed to the high microplastic concentration across food levels. However, this effect was slight when mussels were exposed to the high-food treatment. Surprisingly, mussels exposed to high-microplastics in the low feeding group was slightly higher than the mussels acclimated to high-food only. This is intriguing because digestion and absorption are energy-dependent processes and mussels are consummate energy conservers due to unpredictable energetic threats related to the highly variable and stressful coastal environment. Any anthropogenic disturbances to energy reserves of mussels in nature could affect growth, fitness, survival, and downstream ecological processes. To this point, Van Cauwenberghe and Janssen (2014) [41] revealed higher energy consumption (mitochondrial activity) in plastic-exposed mussels compared to control organisms. Interestingly, Détrée and Gallardo-Escárate (2017) [42] observed elevated gene expression of key energy metabolism genes (pyruvate kinase and succinate dehydrogenase) in the digestive gland but not in other tissues of exposed mussels, suggestive of tissue-dependent elevation of carbohydrate oxidation during particle exposure. It is possible that under very high microplastic exposures, amylase synthesis and secretion was upregulated to compensate for extreme food dilution that occurs under these conditions. As such, Bayne et al. (1988) revealed compensatory responses of the gut in *M*. *edulis*: unpredictably high absorption efficiency was observed under suboptimal food-quality conditions [43]. Absorption efficiency is positively integrated with digestive enzyme activity under certain feeding conditions, therefore enhanced digestive enzyme activity might also play a compensatory role in energy balance under suboptimal food-quality conditions. In agreement with this inference, Ibarrola et al. (2000) [44] showed in cockle *Cerastoderma edule*, a steady maintenance of amylase activity across variation in food quality.

These few examples highlight the need for precision-based studies on the effects of food quality on digestive enzyme activity in *M*. *galloprovincialis* in order to fully resolve its nutrient balancing and compensatory strategies. Furthermore, studies of the effects of microplastics on tissues in intertidal bivalves under simulated stressors of the coastal environment, rather than assessments under benign conditions, are also necessary to understand the full scope of environmental-physiological responses. For example, subjecting mussels to microplastics under a simulated tidal-aerial-exposure thermal cycle, similar to the tidal environment mussels experience in nature, may further reveal the complexities of their digestion flexibility. The high concentration of microplastics and short length of this study further limits our understanding of the effects of more moderate perturbations that occur over yearly timescales.

In conclusion, mussels of the genus *Mytilus* are useful bioindicators of microplastic pollution in the ocean. Our results indicate that enzyme activity of amylase may be detrimental to digestive gland function, but only under certain food regimes. However, more studies that examine a wider scale of nutrition, microplastic contamination, and time under varied environmental conditions are necessary to understand how mussels will cope in the future. Furthermore, simultaneous measurements of additional physiological processes and enzymes are necessary to gain an integrated understanding of digestive flexibility of mussels in the face of anthropogenic perturbations. Importantly, an examination of a range of enzymes including other carbohydrases, lipases, and proteases is necessary to fully understand impacts on digestive gland function in *M*. *galloprovincialis*. In this context Wang (2020) observed negative effects on protease and lipase activity in *M*. *coruscus* [35]. Finally, studies of microplastic effects on gill and digestive gland should be conducted in series so that mechanisms of the effects can ultimately be revealed.

Our findings are supported by Chae & An (2020) [45], who found it is likely that in mussels, a positive relationship exists between food abundance and the retainment of microplastics in laboratory conditions. In addition, microorganisms can adhere to microplastics [46] thereby making these particles potential vectors of organic nutrients to the digestive gland. In the present study we showed that *M*. *galloprovincialis* retained the ability to acquire nutrients despite coming into contact with this harmful pollutant, suggestive that mussels are more resilient than previously hypothesized [25]. Evaluation of the effects of microplastic exposure on physiological performance in mussels is an emerging field. The challenge for clinicians will be establishing consistent controls between experiments for factors such as species, microplastic type, exposure duration, food type and level, temperature, and emergence/submergence cycles.

## Supporting information

S1 File Amylase activity and *HSP70B2* gene expression. (XLSX)
