# Correction: The observation of starch digestion in blue mussel *Mytilus galloprovincialis* exposed to microplastic particles under varied food conditions

**DOI:** 10.1371/journal.pone.0305981

**Published:** 2024-06-18

**Authors:** The PLOS ONE Staff

The first author’s name is spelled incorrectly. The correct name is: CJ O’Brien. The correct citation is: O’Brien CJ, Hong HC, Bryant EE, Connor KM (2021) The observation of starch digestion in blue mussel Mytilus galloprovincialis exposed to microplastic particles under varied food conditions. PLoS ONE 16(7): e0253802. https://doi.org/10.1371/journal.pone.0253802.

There is an error in affiliation 2 for authors Helen C. Hong, Emily E. Bryant and Kwasi M. Connor. The correct affiliation 2 is: Department of Ecology and Evolutionary Biology, University of California, Irvine, California, United States of America.

The publisher apologizes for the errors.
